# Targeting mutant *VHL-*gene results in therapy response in metastatic clear cell carcinoma of rete testis

**DOI:** 10.1016/j.eucr.2026.103376

**Published:** 2026-02-16

**Authors:** Michael Karl Melzer, Angelika Mattigk, Verena I. Gaidzik, Nadine Therese Gaisa, Julia Schwenke, Friedemann Zengerling

**Affiliations:** a Department of Urology and Pediatric Urology, Ulm University Hospital, Ulm, Germany; b Department of Internal Medicine III, Ulm University Hospital, Ulm, Germany; c Center of Personalised Medicine, Ulm University Hospital, Germany; d Department of Pathology, Ulm University Hospital, Germany

## Abstract

Epididymal and rete testis tumors are exceedingly rare. We report a 69-year-old male who presented with progressive swelling of the right testis/epididymis; histopathology confirmed clear cell carcinoma of the rete testis. Staging with FDG-PET-CT revealed bilateral pulmonary metastases. Subsequent gene panel sequencing identified a pathogenic variant of *VHL* and a likely pathogenic variant of *TP53*. Off-label treatment with belzutifan was initiated. Regression of the pulmonary lesion with ongoing complete remission at 12 months of follow-up was observed. This case highlights the importance of molecular profiling for personalized therapy in rare tumor entities.

## 1 Introduction

Epididymal/Rete testis tumors are a highly rare disease entity accounting for only 0.03% of all male tumors.^1^ Sarcomas are the most prominent tumors within the epididymis.^2^ Hence, metastatic clear cell carcinoma of the rete testis/epididymis is an exceptionally rare malignancy, with limited cases documented in literature^3^, ^4^, ^5^. Its rarity poses significant diagnostic and therapeutic challenges. Moreover, therapy in metastatic disease is not established. Hence, targeted approaches for personalized treatment are mandatory.

This case report aims to highlight the clinical course and diagnostic intricacies associated with this rare disease.

## 2 Case presentation

**Fig. 1:**
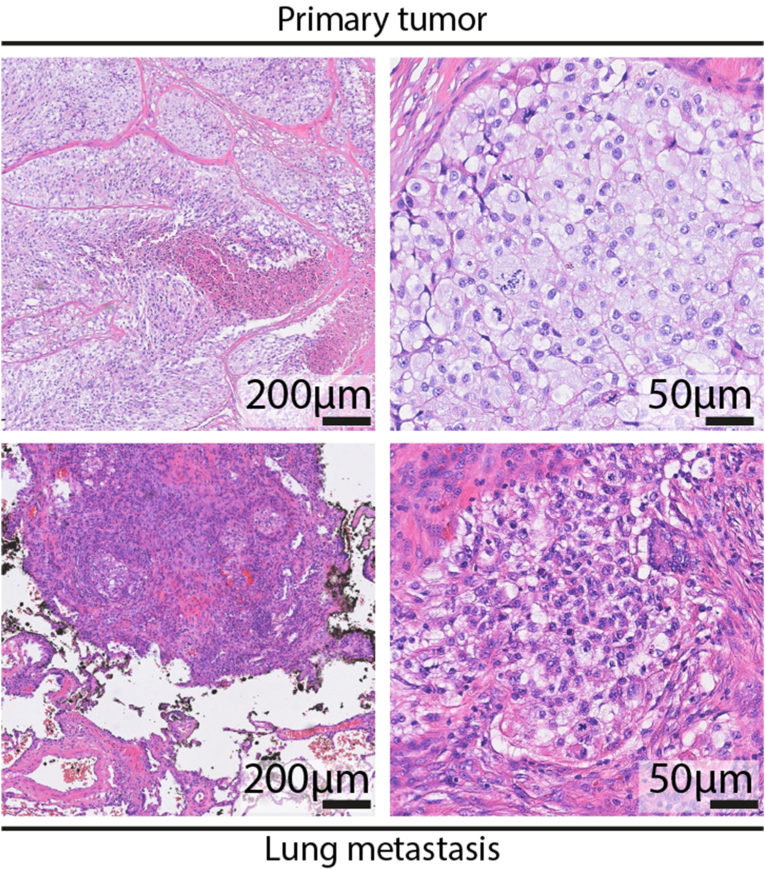
Representative H&E stainings of the clear cell carcinoma aspect of the primary tumor and metastatic site.

**Fig. 2:**
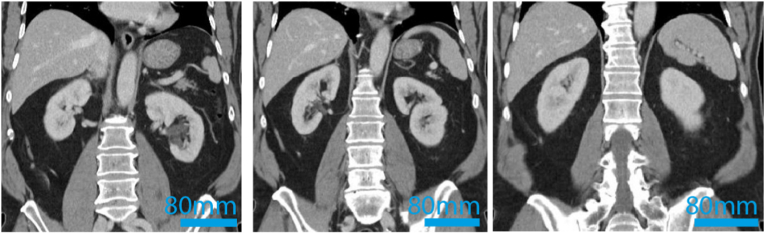
Pre-systemic therapy CT scan of the kidneys ruling out primary tumor of the kidneys.

**Fig. 3:**
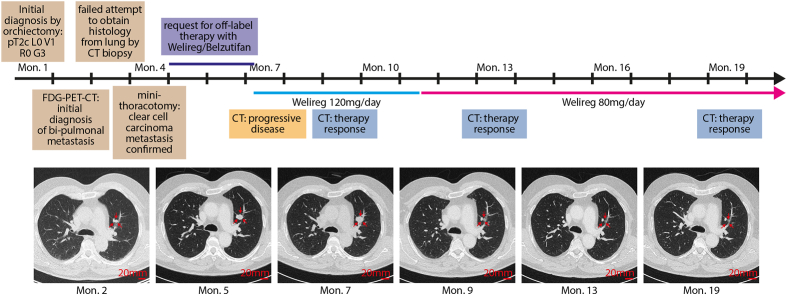
Timeline and exemplary CT-scan-slices for the treatment response.

A 69-year-old male presented with progressive swelling of the right testis/epididymis. Ultrasound imaging was performed with the primary objective of evaluating for testicular cancer. The results of the ultrasound identified a tumor located in the rete testis. This finding played a critical role in guiding further diagnostic steps and therapeutic decision-making for the patient. Hence, epidydimal-orchiectomy was performed by a scrotal approach. Histopathological analysis of the excised tissue confirmed a diagnosis of clear cell carcinoma of the rete testis (Fig. 1). Immunohistochemical analysis of the tumor tissue revealed a distinct expression profile. The tumor cells demonstrated positive staining for PAX-8, epithelial membrane antigen (EMA), carbonic anhydrase IX, CD10, and cytokeratin 7 (CK7). In contrast, the tumor was negative for several other markers, including Calretinin, SALL4, OCT3/4, D2-40, Napsin-A, CA125, estrogen receptor, racemase, GATA3, and CD15. Given the unusual nature of this finding, comprehensive staging was initiated using 18F-fluorodeoxyglucose positron emission tomography/computed tomography (FDG-PET/CT). FDG-PET/CT revealed no evidence of a primary renal tumor, effectively ruling out renal cell carcinoma (RCC) as a potential primary and source of metastasis (Fig. 2, depicting only the CT-sequence for reasons of clarity). Instead, the imaging identified bi-pulmonary metastatic disease, suggesting a systemic spread of the malignancy originating from the rete testis/epididymis (Fig. 3).

Due to technical challenges in obtaining tissue from the lungs via CT-guided biopsy to confirm the metastatic spread, a minithoracotomy was performed. Here, metastatic spreading of the clear cell carcinoma was confirmed (Fig. 1). Concurrently, comprehensive exon panel sequencing including 486 genes (coverage 3000x, Qiagen, sequencing on an Illumina platform) of the primary tumor revealed a somatic class 5 variant in the *VHL* gene (c.286C > T; p.Gln96∗, variant allele frequency [VAF] 30%) and a class 4 variant in *TP53* (c.527G > T; p.Cys176Phe, VAF 29%).^6^

This molecular profile was subsequently discussed at our multidisciplinary molecular and familiar tumor board. Due to the pathogenetic *VHL* variant, treatment with the HIF-2a inhibitor belzutifan (Welireg®) was recommended based on an evidence level m2A. Notably, belzutifan was recently approved by the U.S. Food and Drug Administration for the treatment of von Hippel-Lindau (VHL) disease–associated renal cell carcinoma (U.S. Food and Drug Administration, 2021) based on a prior phase-2 study.^7^

Given that belzutifan is not approved for clear cell carcinoma of the rete testis/epididymis, we have formally requested permission from the patient's health insurance provider to proceed with this off-label therapy. After obtaining approval, treatment was started at a dose of 120 mg per day (7 months after initial diagnosis). Notably, pre-therapy imaging had identified a growing pulmonary metastasis (Fig. 2); during belzutifan treatment, serial imaging demonstrated gradual regression of this lesion, and after one year of follow-up, the overall disease status was near to complete remission with fibrotic residues of the known lung metastasis, only.

**Fig. 4:**
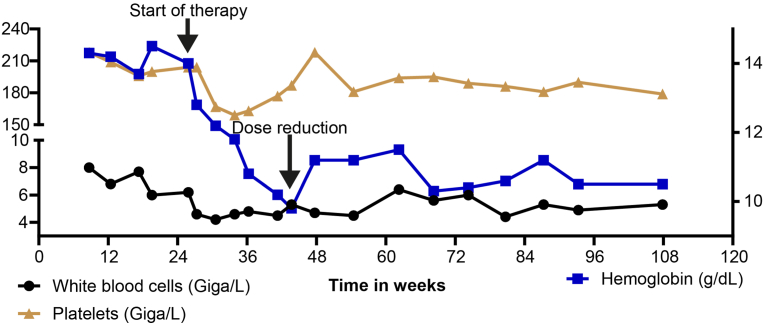
**Laboratory assessment of different hematological parameters**, including hemoglobin (Hb) depicted on the right axis, as well as platelets and white blood cell count depicted on the left axis.

After approximately 4 months of therapy, the dose was reduced to 80 mg per day due to the emergence of side effects. The most prominent adverse effect was mild (grade I) fatigue resulting from anemia, with the patient's hemoglobin levels declining from values exceeding 14 g/dL to approximately 10 g/dL (Fig. 3). Additional laboratory monitoring revealed no significant changes in renal function (creatinine), electrolyte balance (potassium and sodium), or hepatic parameters (AST/ALT, data not shown). A modest decrease in both white blood cell counts and platelet levels was noted (Fig. 4); however, these changes were not associated with any clinically significant sequelae. Importantly, aside from these hematologic toxicity, the patient has not experienced any other side effects that impact overall quality of life.

## 3 Discussion

This case highlights several important considerations in the management of extremely rare malignancies. First, the recent FDA approval of belzutifan for either advanced renal cell carcinoma^8^ or VHL-syndrome associated malignancies (e.g., renal cell carcinoma, central nervous hemangioblastomas, pancreatic neuroendocrine tumors, excluding the presented case) underscores its potential as a tumor-agnostic agent for VHL-altered tumors.^9^ Notably, VHL is associated with a germline variant, whereas the genetic profile of our patient was tested in somatic tumor tissue suggesting that *VHL*-mutant tumors *per se* may benefit from a targeted therapy irrespective of the germline origin. However, germline testing was not conducted in this patient, given the presence of a confirmed somatic mutation.

Second, the off-label use of belzutifan in our case necessitated obtaining prior authorization from the patient's health insurance provider, which delayed the initiation of treatment. This delay underscores the challenges associated with accessing innovative therapies in a timely manner, particularly when used outside of their approved indications. Lastly, the case emphasizes the broader need for molecular targeted therapies in rare cancer entities. As traditional treatment options are often limited or ineffective in these scenarios, comprehensive molecular profiling is crucial to identify actionable targets and guide personalized therapeutic approaches. Overall, this experience supports further exploration and potentially broader regulatory approval of belzutifan in a tumor-agnostic fashion for *VHL*-altered tumors, while also highlighting the systemic challenges that can delay access to promising treatments.

## CRediT authorship contribution statement

**Michael Karl Melzer:** Writing – review & editing, Writing – original draft, Visualization, Validation, Methodology, Investigation, Formal analysis, Data curation, Conceptualization. **Angelika Mattigk:** Writing – review & editing, Supervision, Resources, Data curation, Conceptualization. **Verena I. Gaidzik:** Writing – review & editing, Supervision, Formal analysis. **Nadine Therese Gaisa:** Writing – review & editing, Data curation. **Julia Schwenke:** Writing – review & editing, Visualization, Data curation. **Friedemann Zengerling:** Writing – review & editing, Supervision, Resources, Investigation, Funding acquisition, Data curation, Conceptualization.

## Informed consent

The patient gave full informed written consent for this case report.

## Declaration of generative AI and AI-assisted technologies in the manuscript preparation process

During the preparation of this work the author(s) used ChatGPT (GPT5, OpenAI) and Microsoft Co-Pilot in order to enhance readability of the manuscript text. After using this tool, the authors reviewed and edited the content as needed and take full responsibility for the content of the published article.

## Competing interests

The authors declare no conflict of interest.

## Data Availability

Anonymized data can be partly made available on reasonable requests. Imaging data will not be shared.
